# 535. Evaluation of the BioFire Blood Culture Identification (BCID2) panel for transplant recipients with a bloodstream infection

**DOI:** 10.1093/ofid/ofac492.588

**Published:** 2022-12-15

**Authors:** Carlo Foppiano Palacios, David Peaper, Maricar F Malinis, Maricar F Malinis, Sarah Perreault, Elizabeth A Cohen, Joshua Vogel, Marwan M Azar

**Affiliations:** Yale University, New Haven, Connecticut; Yale University, New Haven, Connecticut; Yale University, New Haven, Connecticut; Yale University, New Haven, Connecticut; Yale New Haven Hospital, New Haven, Connecticut; Yale New Haven Hospital, New Haven, Connecticut; Yale University, New Haven, Connecticut; Yale University, New Haven, Connecticut

## Abstract

**Background:**

In patients with bloodstream infections (BSI), the BioFire blood culture identification (BCID2) multiplex PCR panel is associated with decreased time to organism identification and antimicrobial susceptibility results needed to guide optimal therapy. While the performance of BCID2 has been evaluated in the general population, data for transplant recipients are limited. We sought to identify the utility of the BCID2 panel in transplant recipients.

**Methods:**

A retrospective chart review was conducted to identify all solid organ recipients (SOTR) and bone marrow transplant recipients (BMTR) within 2 years of transplantation with BSI for whom BCID2 was performed from 06/2021 to 01/2022. Demographic and microbiological data were collected. Positive blood cultures for the same patient and same organism(s) occurring within 14 days of the initial test were considered a single BSI event. Descriptive statistics were performed.

**Results:**

A total of 29 transplant recipients with 45 positive blood cultures underwent BCID2 testing. Mean age was 54 years, 69% were BMTR and 53% of cultures were from peripheral sites. A total of 51 organisms were recovered from 45 blood cultures. Organisms identified by blood cultures included Enterobacterales (45%), *Enterococcus* spp (20%), *Candida* spp (8%), non-Enterobacterales gram negative rods (14%), *Streptococcus* spp (8%), and other gram positives (5%). No anaerobic organisms were isolated. BCID2 did not detect 7/51 (14%) organisms identified by blood cultures including monomicrobial (n=6/39) and polymicrobial (n=1/6) cultures. All 7 organisms not identified by BCID2 were not in the BCID2 database. All occurred in BMTR; 4 were considered pathogenic and treated with antimicrobials vs. 3 contaminants (Table 1). BCID2 detected CTX-M or Van A/B in all 15 samples with ceftriaxone (n=9) or vancomycin resistance(n=6).

**Figure:**
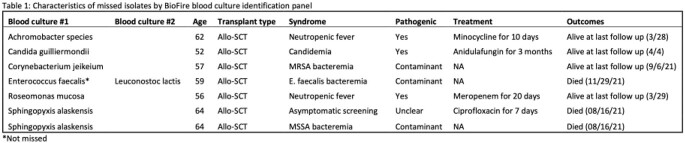

**Conclusion:**

In transplant recipients, BCID2 detected 86% of organisms and 100% of resistance markers identified by conventional testing. All 7 (14%) missed cases involved off-target organisms, of which 4 were considered pathogenic. BCID2 is a useful tool for BSI detection in transplant recipients, but providers should consider the possibility of off-target pathogens when clinically appropriate.

**Disclosures:**

**David Peaper, MD**, Tangen Biosciences: Stocks/Bonds **Maricar F. Malinis, MD**, Aicuris: Primary Investigator of Clinical Trial at Yale Site|Takeda: Primary investigator of clinical trial at Yale Site **Maricar F. Malinis, MD**, Aicuris: Primary Investigator of Clinical Trial at Yale Site|Takeda: Primary investigator of clinical trial at Yale Site.

